# Critical sources of bacterial contamination and adoption of standard sanitary protocol during semen collection and processing in Semen Station 

**DOI:** 10.14202/vetworld.2015.631-635

**Published:** 2015-05-21

**Authors:** Chandrahas Sannat, Ajit Nair, S. B. Sahu, S. A. Sahasrabudhe, Ashish Kumar, Amit Kumar Gupta, R. K. Shende

**Affiliations:** 1 Department of Veterinary Microbiology, College of Veterinary Science & A.H., Chhattisgarh Kamdhenu Vishwavidyalaya, Anjora, Durg, Chhattisgarh, India; 2 Central Semen Station, Livestock Development Department, Government of Chhattisgarh, Anjora, Durg, Chhattisgarh, India

**Keywords:** bacterial contamination, critical sources, environment, laboratory equipments, Semen Station

## Abstract

**Aim:**

The present investigation was conducted to locate the critical sources of bacterial contamination and to evaluate the standard sanitation protocol so as to improve the hygienic conditions during collection, evaluation, and processing of bull semen in the Semen Station.

**Materials and Methods:**

The study compared two different hygienic procedures during the collection, evaluation and processing of semen in Central Semen Station, Anjora, Durg. Routinely used materials including artificial vagina (AV) inner liner, cone, semen collection tube, buffer, extender/diluter, straws; and the laboratory environment like processing lab, pass box and laminar air flow (LAF) cabinet of extender preparation lab, processing lab, sealing filling machine, and bacteriological lab were subjected to bacteriological examination in two phases of study using two different sanitary protocols. Bacterial load in above items/environment was measured using standard plate count method and expressed as colony forming unit (CFU).

**Results:**

Bacterial load in a laboratory environment and AV equipments during two different sanitary protocol in present investigation differed highly significantly (p<0.001). Potential sources of bacterial contamination during semen collection and processing included laboratory environment like processing lab, pass box, and LAF cabinets; AV equipments, including AV Liner and cone. Bacterial load was reduced highly significantly (p<0.001) in AV liner (from 2.33±0.67 to 0.50±0.52), cone (from 4.16±1.20 to 1.91±0.55), and extender (from 1.33±0.38 to 0) after application of improved practices of packaging, handling, and sterilization in Phase II of study. Glasswares, buffers, and straws showed nil bacterial contamination in both the phases of study. With slight modification in fumigation protocol (formalin @600 ml/1000 ft^3^), bacterial load was significantly decreased (p<0.001) up to 0-6 CFU in processing lab (from 6.43±1.34 to 2.86±0.59), pass box (from 12.13±2.53 to 3.78±0.79), and nil bacterial load was reported in LAFs.

**Conclusion:**

Appropriate and careful management considering critical points step by step starting right from collection of semen to their processing can significantly minimize bacterial contamination.

## Introduction

Economic pressure with the aim to achieve high milk production with minimal inputs has laid down the development of new technologies, i.e., artificial insemination (AI). The use of frozen semen is one of the spectacular developments in modern day AI programs. The contamination of fresh and preserved semen poses a great risk to the successful breeding program as it may lead to rapid decline in sperm motility [1] and subsequent fertility [2]. The microbes, because of their ubiquitous presence have ample chance to contaminate semen during collection, processing and preservation. Bacterial contamination of semen may occur at any time right from collection through the various steps involved in preparing frozen straws. Artificial vagina (AV), glasswares, semen extender and laboratory environment are some of the common sources that may contribute to the bacterial load of semen during processing [3].

Application of good sanitation practice in Semen Station is the key issue to improve the quality of cryopreserved semen. As per the fundamental principles of critical hazard analysis [4], identification of critical control points are essential to establish monitoring system and corrective action. Few attempts have already been made to identify and locate critical control points at the level of semen collection and processing [5-7]. Each Semen Station is following a protocol, which is standard for their respective situation. However, it is essential to revalidate semen collection and processing protocol at regular interval to achieve the standard. Though zero risk does not exist in the biological world, but it should be as close to it as possible.

Present study was therefore undertaken to identify the common sources of bacterial contamination across a series of cryopreservation process of semen; and to evaluate the impact of two different sanitation protocols in Semen Station so as to improve the hygienic status of semen.

## Materials and Methods

### Ethical approval

No ethical approval was necessary to pursue this research work.

### Samples and experimental design

Present study was conducted at Central Semen Station, Anjora, Durg, Chhattisgarh, India. Present study compared two different sanitation protocols during collection and processing of semen in Semen Station. Materials routinely used for preparation of AV and frozen semen straw; and the laboratory environment, which could be the possible source of bacterial contamination during semen collection and processing were identified and categorized as AV equipments including AV inner liner, cone, semen collection tube; and other glasswares; buffer, extender/diluter, straws, and laboratory environment including processing lab, pass box, and laminar air flow (LAF) cabinet of extender preparation lab, processing lab, sealing filling machine, and bacteriological lab. Above items/environment were subjected to bacteriological analysis during two different phases of study, i.e., Phase I (during first 3 months of study) and Phase II (next 3 months of study). In first phase of the study, routine standard sanitation protocol [8-9] was followed in Semen Station. To improve the hygienic condition, slight modifications [10-11] were made in sanitation protocol of Phase II study.

### Sanitation protocol in phase I

#### Personnel hygiene

This included the use of sterilized apron, cap, and hands. Aprons were sterilized by autoclaving (at 121°C and 15 lbs pressure for 15 min) and hands were disinfected with 0.1% savlon (Cetrimide and Chlorhexidine Gluconate Solution^®^ Novartis).

#### Sterilization of glass wares

Glass wares were sterilized by hot air oven method at 160°C for 1 h after covering the open end of dried glass wares.

#### AV equipments

AV cylinders and cones were sterilized in AV sterilizer (at 100°C atmospheric pressure).

#### Water and buffer

Sterile Mlli-Q purified water was used for the preparation of buffer. Both water and buffer were sterilized by autoclaving at 121°C and 5 lbs pressure for 20 min.

#### Sanitation of environment

Laboratory environment including processing lab, LAF, apron, and laboratory footwear cabinets were sterilized with ultraviolet rays (2470 A°) for 8 h prior to the commencement of work. Besides, processing lab was being sterilized twice a week with humidifier using cold fumigant formalin (Formaldehyde Solution 37-41% W/V^®^ Qualigens) @ 500 ml/1000 ft^3^ for 2 h. Furthermore, all the work platform and incubator used for keeping AVs were disinfected with 70% ethyl alcohol (Ethanol Absolute 99.9%^®^ SDFCL) on a routine basis.

### Sanitation protocol in phase II

Quite a few attempts have been made to improve the quality of AV equipments and laboratory environments in next 3 months of study. Slight modifications were made in protocol of sterilization and packaging of AVs, sterilization of LAF cabinets, pass box, and fumigation of processing lab, as mentioned below:

Fully assembled AVs (AV cylinder, cone, and semen collection tube) were sterilized in AV sterilizer. Incubators used for keeping AVs; LAF cabinets and pass box were being sterilized by fumigation on weekly basis using potassium permanganate (Potassium Permanganate AR^®^ SDFCL) and formalin in combination @ 150 g of potassium permanganate to 280 ml formalin per 1000 ft^3^ [10] and routinely disinfected with 70% ethanol before and after work [9]. To minimize exposure with the environment, open end of sterile AV was covered with sterile aluminum foil and removed at the time when the bull was ready for ejaculation. 0.1% savlon was used as hand disinfectant by persons involved in AV preparation. Hand gloves were being changed after assembly of each AV. To overcome higher bacterial load in a laboratory environment, fumigation was performed with increased concentration of formalin @ 600 ml/1000 ft^3^ for 2 h [11].

### Determination of bacterial load in buffer, extender, straws, AV equipments, and other glasswares

Sampling of above materials was done according to the protocol described by Brown *et al*. [12]. AV equipments, glasswares, and straws were rinsed/infused with sterile distilled water and kept for 2-3 min. Further, the bacterial load was measured using standard plate count (SPC) method as described by Shukla *et al*. [13]. 1 ml each of buffer, diluter and rinse collected from AV equipments, straws and glasswares were spread on separate SPC agar plates (Plate Count Agar^®^ Hi-Media) and then incubated for 72 h at 37°C. Colonies produced in SPC agar plates were counted and expressed as colony forming unit (CFU).

### Determination of bacterial load in laboratory environment

Passive air sampling technique as described by Pasquarella *et al*. [14] was used to assess microbial air contamination. SPC agar plates with a diameter of 9 cm were exposed in different locations of laboratory i.e., center of processing lab, pass box, and LAF cabinet of processing lab, extender preparation lab, bacteriological lab, and sealing filling machine, according to the 1/1/1 scheme (for 1 h, 1 m above the floor, about 1 m away from walls or any major obstacles). Then, SPC agar plates were incubated at 37°C for 72 h. Colonies produced in SPC agar plates were counted and expressed as CFU/m^2^/h.

### Data recording and statistical analysis

Data were expressed as means (±standard error of the mean) CFU and analyzed by applying general linear model for factorial experiments using SPSS computer software package (Version 16.0.0.247^©^ 2007). Duncan’s multiple range tests were done to make specific treatment comparisons for values that were found significant by ANOVA.

## Results and Discussion

Likewise present study, Bhakat *et al*. [15], Perumal *et al*. [16], and Miller and Salisbury [17] also reported AV equipments, buffer, extender, and laboratory environment as the potential sources of bacterial contamination at the level of semen collection and processing. Impact of hygienic practices in the laboratory is routinely assessed with the help of SPC methods used in present study [12, 18].

Bacterial load observed in AV equipments and media during two different phases of the study are shown in Tables-1 and 2. Highly significant (p<0.001) variation in bacterial load was reported between two phases of the study. No bacterial contamination was reported in buffer, straws, semen collection tubes and other glasswares in both the phases of study, which positively justified the method of sterilization used and hygienic practices adapted in the laboratory. On contrary, Brown *et al*. [12] reported bacterial load of 5 (0-35) CFU in the buffer and 0-2 CFU in test tubes, even after sterilization by autoclaving and hot air oven method. Though the microwave oven method is found superior over hot air oven method for sterilization of glasswares [19], but the present study reported nil bacterial loads in glass wares after sterilization by hot air oven method only. In the first phase of the present study, a bacterial load of 1-2, 1-3 and 1-7 CFU per plate was observed in extender, the inner liner of AV and cone, respectively. However, after proper packaging, handling and sterilization of AV, count was significantly (p<0.001) reduced to 0-1 CFU (AV liner), 1-3 CFU in cone and nil in extender, which was supported by findings of Thibier and Guerin [20]. In contrast to above finding, Brown *et al*. [12] observed extremely higher count of 320 (0-3100) CFU per plate, contributing more than 50% of total count in about 9% of semen samples.

**Table-1:** Bacterial load in AV equipments, buffer and extender in two different phases of study.

Name of material	Number of samples	Phase I (bacterial load as CFU/plate)		Phase II (bacterial load as CFU/plate)		Significant level
	
		Range (minimum-maximum)	Mean±SEM	Range (minimum-maximum)	Mean±SEM	
AV liner	36	1-3	2.33^a^±0.67	0-1	0.50^b^±0.52	0.001
Cone	36	0-7	4.16^a^±1.20	1-3	1.91^b^±0.55	0.001
Semen collection tube	36	0	0	0	0	
Other glassware (flasks, beakers, pipettes etc.)	36	0	0	0	0	
Buffer	14	0	0	0	0	
Extender	14	0-2	1.33^a^±0.38	0	0^b^	0.001
Straws (10 from each new batch)	20	0	0	0	0	

Values with different superscript in column 4 and 6 differ highly significantly (p<0.001), SEM=Standard error of mean, CFU=Colony forming unit, AV=Artificial vagina

**Table-2:** Bacterial load in different locations of the semen production laboratory during two different phases of the study.

Location	Number of exposure to environment	Phase I (bacterial load as CFU/m²/h)		Phase II (bacterial load as CFU/m²/h)		Significant level
	
		Range (minimum-maximum)	Mean±SEM	Range (minimum-maximum)	Mean±SEM	
Center of processing lab	23	2-15	6.43^a^±1.34	0-6	2.86^b^±0.59	0.001
Pass box	23	6-29	12.13^a^±2.53	0-12	3.78^b^±0.79	0.001
LAF (processing lab)	23	0-1	0.30^a^±0.06	0	0^b^	0.005
LAF (bacteriological lab)	23	0	0	0	0	-
LAF (extender preparation lab)	23	0-2	0.39^a^±0.08	0	0^b^*	0.005
LAF (sealing filling machine)	23	0-3	0.57^a^±0.11	0	0^b^	0.005

Values with different superscript in column 4 and 6 differ highly significantly at significant level 0.001 and 0.005, SEM=Standard error of mean, CUF=Colony forming unit, LAF=Laminar air flow

Laboratory environment including carpets and ceiling generating high humidity inside the closed room are the wide sources of the bacterial population, which adversely affects the quality of materials being exposed [21-23]. In present investigation, LAF of bacteriological lab showed nil bacterial load in both phases of study, whereas that of processing lab, sealing filling machine, and extender preparation lab yielded few bacterial load of 0-1, 0-3, and 0-2 CFU/m²/h in first phase. However, higher bacterial load was recorded in center of processing lab (2-15 CFU/m²/h) and pass box (6-29 CFU/m²/h) during first phase, which is in agreement with findings of Brown *et al*. [12] and Napoli *et al*. [22] who reported 19 and 25 CFU/m²/h in laboratory environment and operation theater, respectively. Bacterial load in a laboratory environment from various locations of the semen production lab in the present investigation was lower than the permissible limit of 20-50 CFU/m²/h [8]. It was observed that after application of fumigation in LAFs and pass box; and slight modification of fumigation protocol in processing lab, bacterial load was significantly (p<0.001) reduced to 0-12 CFU/m²/h (pass box), 0-6 CFU/m²/h (processing lab environment) and nil (p<0.005) in LAF, which is also supported with the report of Arora [8].

No count for AV equipments and diluter has been suggested as they are to be used after strict sterilization [6], but even under careful conditions semen may get contaminated at the time of collection or subsequent handling/packaging. Furthermore, the aim of obtaining sterile semen is almost unachievable. However, the use of sterile equipments and environment helped to prevent further contamination of the semen sample.

## Conclusion

The procedure involving part human and part mechanized operations can lead to bacterial contamination at various levels right from collection to preparation of semen straws. Present study reported AV equipments and laboratory environment as the potential sources of bacterial contamination. Bacterial load in AV equipments and laboratory environments during two different sanitary protocol in the present study differed significantly (p<0.001). Thus, for standardization of best method, establishment of control points are essential, and the quality control while preparing frozen semen should be done at each and every steps starting from preparation of AV, glass wares or preparation of media and extender. Protocols therefore required to be revalidated at regular intervals to achieve the standard quality semen.

## Authors’ Contributions

CS and AN designed the experiment. Media preparation, sample collection, and bacteriological analysis were performed by CS, AK, AKG, and RKS under the supervision of SAS. Sterilization of equipments, environment, and other substances were performed by SBS, CS, and AN. Statistical analysis was done by CS and AN under the supervision of SAS. Manuscript preparation was reviewed and edited by all authors.
